# Supplementary material for: Synergistic effects of sulopenem in combination with cefuroxime or durlobactam against Mycobacterium abscessus
Source: mBio. 2024 May 14;15(6):e00609-24. doi: 10.1128/mbio.00609-24 (PMC11237399; doi:10.1128/mbio.00609-24)

Supplement Table 1. MIC_{50}s against clinical *Mab* isolates *Mab* 122 and *Mab* 686 Evaluating sulopenem (SUL), cefuroxime (CXM), combined sulopenem with 4 µg/ml cefuroxime, and addition of β-lactamase inhibitors (dudrlobactam (DUR) + sulbactam (SULB) or 4 µg/ml of avibactam (AVI).

	MIC_{50} (µg/ml)			
**Antibiotics (µg/ml)**	**Alone**	**+ DUR+SULB (1:1)**	**+ DUR+SULB (1 µg/ml)**	**+ AVI (4 µg/ml)**
against Mab 122				
DUR+SULB (1:1 ratio)	8	-	-	-
AVI	>128	-	-	-
SUL	0.5	0.5	0.5	1
CXM	4	1	2	4
SUL + CXM (2 µg/ml)	≤0.0625	≤0.0625	≤0.0625	≤0.0625
SUL + CXM (4 µg/ml)	≤0.0625	≤0.0625	≤0.0625	≤0.0625
against Mab 686				
DUR+SULB (1:1 ratio)	8	-	-	-
AVI	>128	-	-	-
SUL	2	1	1	2
CXM	8	1	2	8
SUL + CXM (2 µg/ml)	1	0.5	≤0.0625	≤0.0625
SUL + CXM (4 µg/ml)	0.25	≤0.0625	≤0.0625	≤0.0625

Supplement Fig. 1. Intact-protein UPLC-IM-MS analysis reveals that sulopenem binds to Ldt_{Mab2}, Ldt_{Mab4}, DDC, and PBP B within a 5-min. Binding of sulopenem to Ldt_{Mab3} exhibits a binding time exceeding 2 hours. On the other hand, cefuroxime shows binding only towards Ldt_{Mab2} and PBP B. The depicted figure illustrates the interactions, where the black bars represent apo-LDTs, DDC, and PBP B; the red bars indicate the LDTs, DDC, and PBP B-sulopenem adducts, and the purple bars represent the LDTs, DDC, and PBP B-cefuroxime adducts.

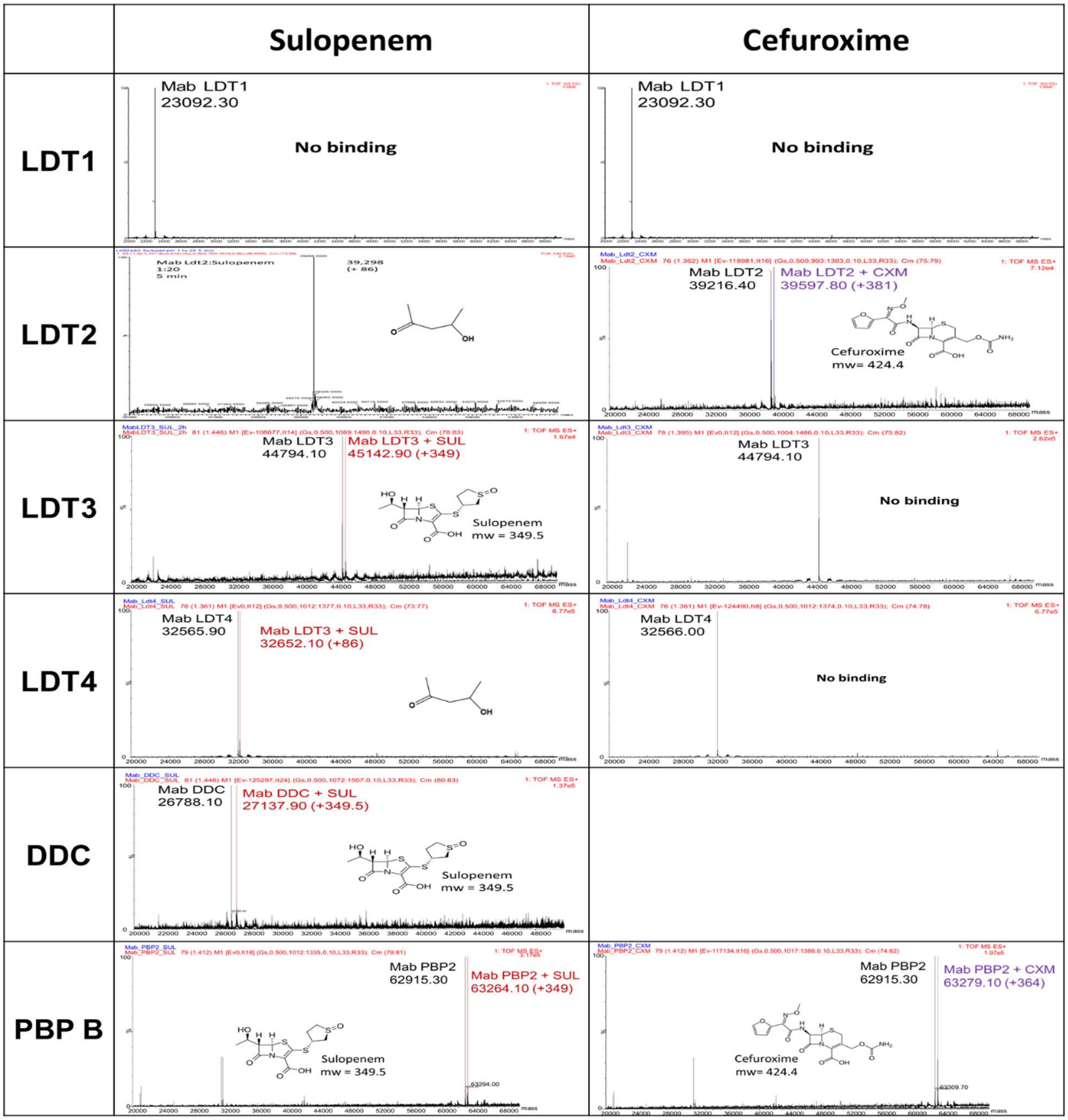

Supplement Fig. 2: Time-kill curves of sulopenem (A) in monotherapy, cefuroxime (CXM) in monotherapy (B), the combination of sulopenem and CFX (C and D), and sulopenem in the presence of BLIs (avibactam; AVI and durlobactam; DUR) (E) against clinical isolate *Mab* 122.

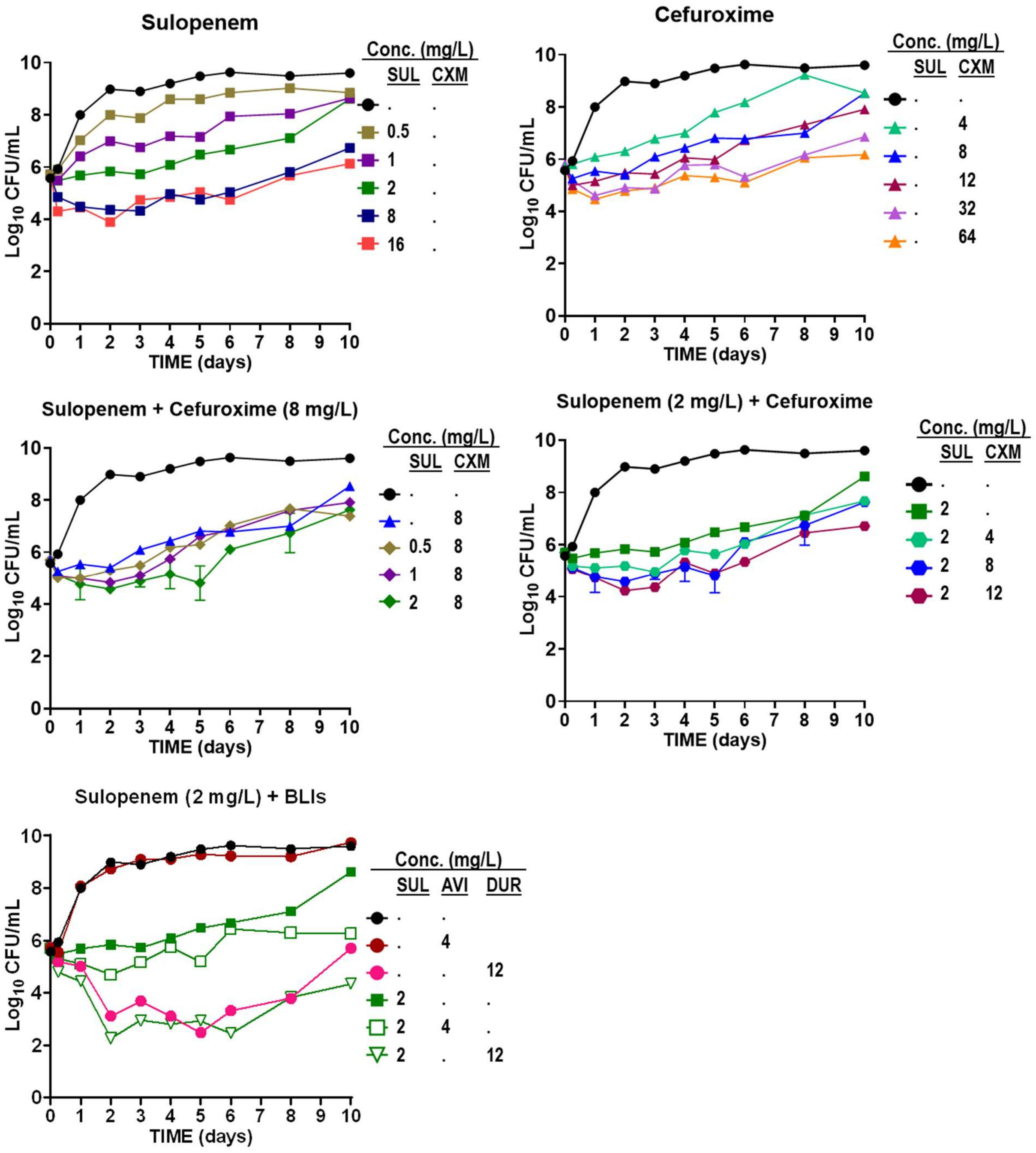

Supplement Fig. 3: Time-kill curves of sulopenem (A) in monotherapy, cefuroxime (CXM) in monotherapy (B), the combination of sulopenem and CXM (C and D), and sulopenem in the presence of BLIs (avibactam; AVI and durlobactam; DUR) (E) against clinical isolate *Mab* 686.

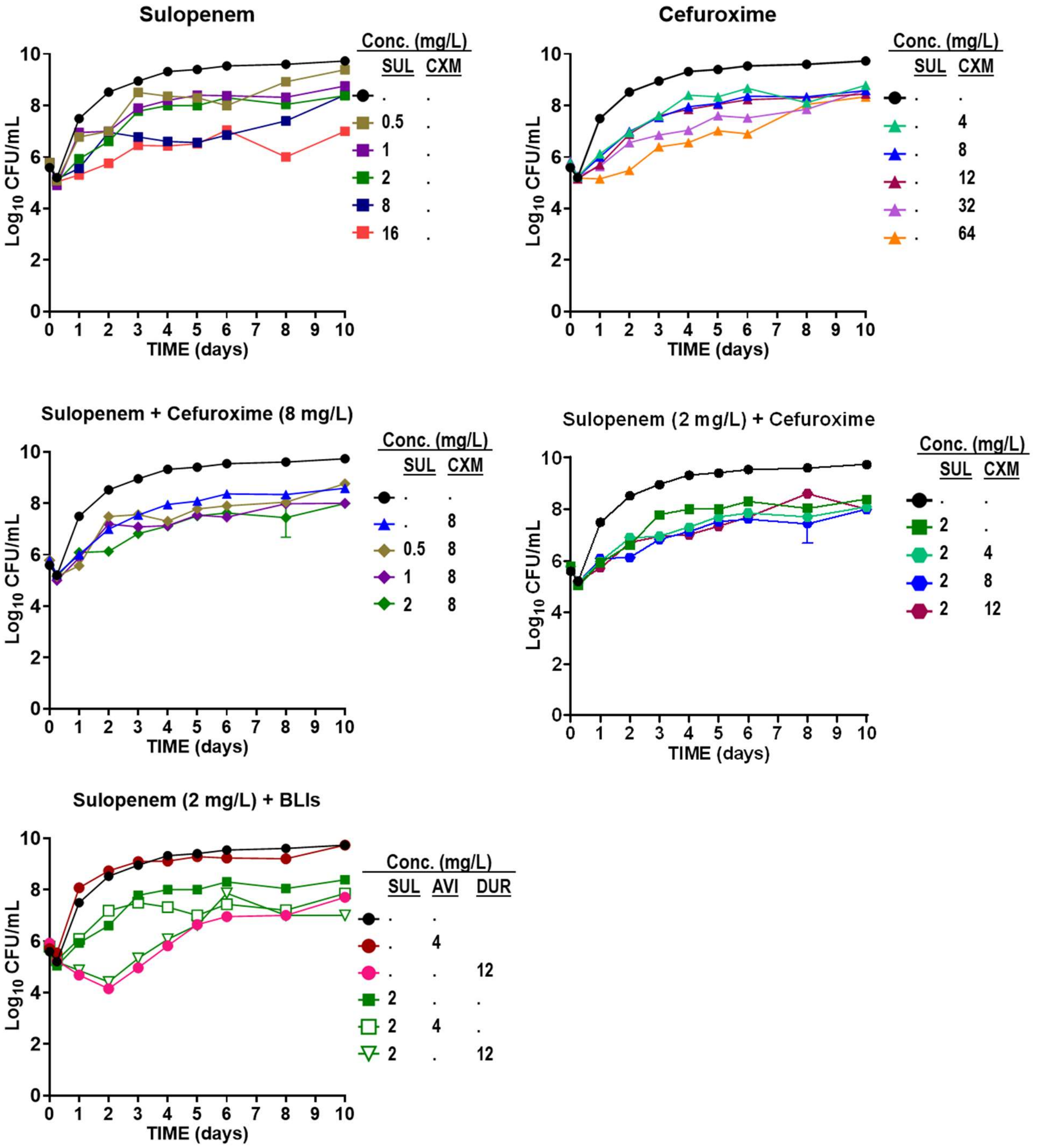

Supplement Fig.4: Ldt$_{Mab2}$ (A, C) *vs.* Ldt$_{Mab3}$ (B, D) active site variability and shape. The deletion and insertion into the loops of the active site are colored yellow and red for sequnce alingnment representation (F).

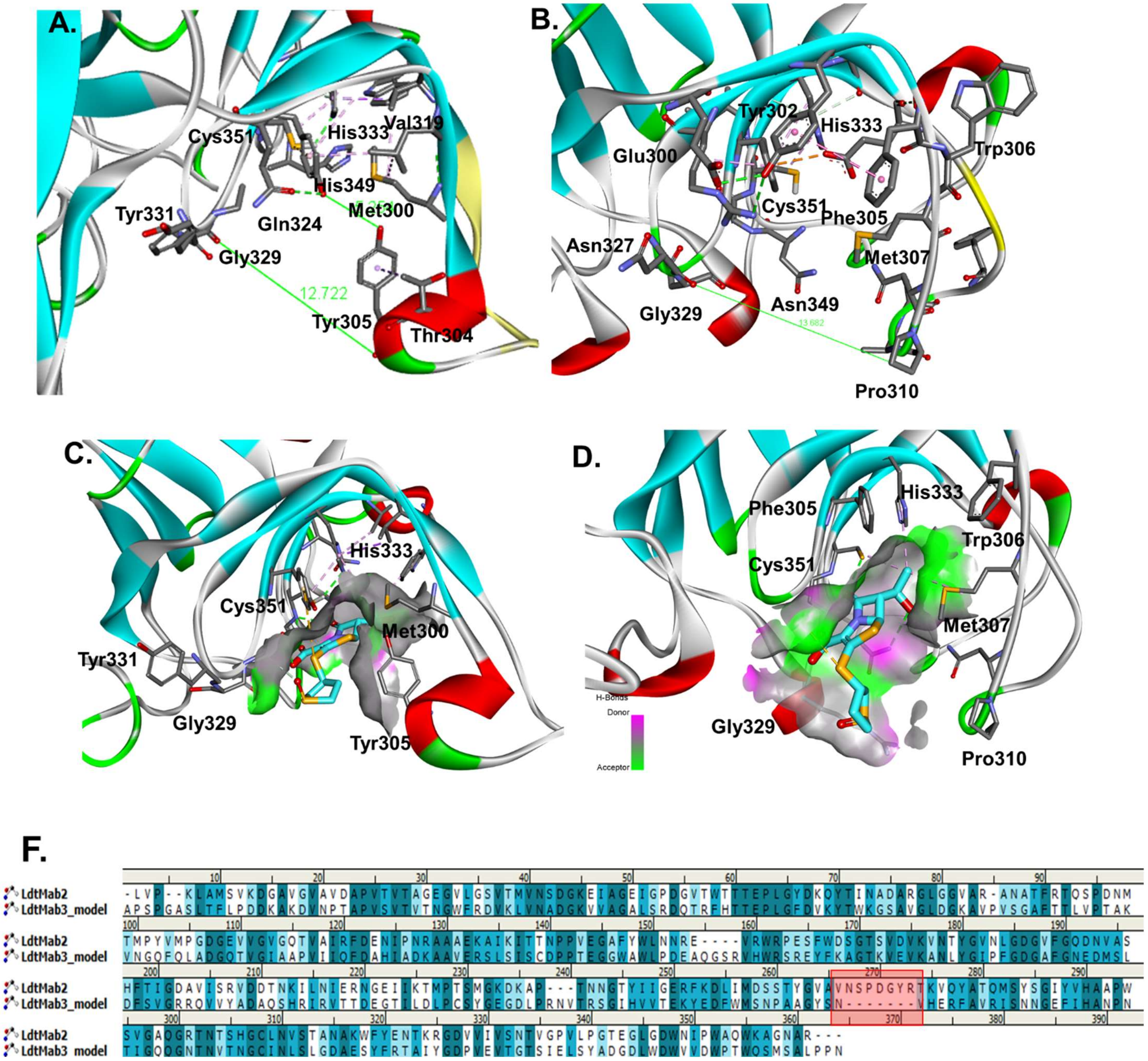

Supplement: Supplemental material — Table S1 and Fig. S1 to S4. [file mbio.00609-24-s0001.pdf]
